# Common-path interferometric label-free protein sensing with resonant dielectric nanostructures

**DOI:** 10.1038/s41377-020-0336-6

**Published:** 2020-06-02

**Authors:** Isabel Barth, Donato Conteduca, Christopher Reardon, Steven Johnson, Thomas F. Krauss

**Affiliations:** 1 Department of Physics, University of York, YO10 5DD York, UK; 2 Department of Electronic Engineering, University of York, YO10 5DD York, UK

**Keywords:** Biophotonics, Imaging and sensing, Optical sensors, Photonic crystals, Nanophotonics and plasmonics

## Abstract

Research toward photonic biosensors for point-of-care applications and personalized medicine is driven by the need for high-sensitivity, low-cost, and reliable technology. Among the most sensitive modalities, interferometry offers particularly high performance, but typically lacks the required operational simplicity and robustness. Here, we introduce a common-path interferometric sensor based on guided-mode resonances to combine high performance with inherent stability. The sensor exploits the simultaneous excitation of two orthogonally polarized modes, and detects the relative phase change caused by biomolecular binding on the sensor surface. The wide dynamic range of the sensor, which is essential for fabrication and angle tolerance, as well as versatility, is controlled by integrating multiple, tuned structures in the field of view. This approach circumvents the trade-off between sensitivity and dynamic range, typical of other phase-sensitive modalities, without increasing complexity. Our sensor enables the challenging label-free detection of procalcitonin, a small protein (13 kDa) and biomarker for infection, at the clinically relevant concentration of 1 pg mL^−1^, with a signal-to-noise ratio of 35. This result indicates the utility for an exemplary application in antibiotic guidance, and opens possibilities for detecting further clinically or environmentally relevant small molecules with an intrinsically simple and robust sensing modality.

## Biosensors: Nanostructures team up to turn down the noise

Portable devices that spot biomarkers in a patient’s bloodstream stand to benefit from a photonic microchip that can achieve ultra-low detection limits. Isabel Barth from the University of York in the United Kingdom and colleagues designed a biosensor based on guided-mode resonances, a phenomenon that uses laser light to switch on strong resonant signals inside nanoscale gratings. The researchers improved this technology by simultaneously exciting two resonant modes with a low-cost laser diode. This approach enables sensing of protein-antibody binding induced refractive index changes using relative phase differences between the two modes, minimizing the impact of noise due to outside sources including mechanical vibrations. The team demonstrated label-free detection of procalcitonin, a key biomarker for bacterial infections, at picogram/milliliter scales, which could, for example, be relevant in the context of bacterial co-infections of COVID-19 patients.

## Introduction

The challenge of developing a point-of-care (POC) diagnostic device lies in the need for high-sensitivity detection of low concentrations of biomarkers in a low-cost platform that can be operated by nonspecialists. Achieving this goal will provide new opportunities for improving the delivery and speed of diagnostic tests, and underpin the move toward personalized and stratified treatment. For example, procalcitonin (PCT) is an inflammatory biomarker of increasing interest that can facilitate the differentiation between bacterial and viral infection^1^. A highly sensitive POC device capable of detecting PCT could thus support rational and targeted use of antibiotics, and a reduction in the spread of antimicrobial resistance^2^.

Photonic sensors based on evanescent field refractive index sensing have proven to be well suited for such high-sensitivity and label-free biosensing; among these sensors, interferometric approaches provide particularly low limits of detection (LODs). Examples of high-performance interferometric systems include integrated Mach–Zehnder^3^ and Young interferometers^4^ based on planar optical waveguides, which show state-of-the-art bulk LODs on the order of 10^−8^ refractive index units (RIU). In the context of POC devices, the disadvantage of these approaches is the need for precise light coupling into waveguides, which implies high-precision angular and spatial alignment^5^. In contrast, sensor modalities using out-of-plane excitation instead of waveguide coupling can offer a higher level of simplicity and tolerance.

An interferometric version of the well-known surface plasmon resonance (SPR) modality was first introduced in 1997 by Kabashin and Nikitin^6^, demonstrating an improvement in LOD of two orders of magnitude (to 4 × 10^−8^ RIU) compared with more traditional noninterferometric angular interrogation. The downside of this method, however, is the trade-off between sensitivity and dynamic range^7,8^. For example, interferometric SPR can achieve LODs of Δ*n* ≈ 10^−8^, but this sensitivity is only achieved over 5 × 10^−5^ RIU, unless advanced designs are used that are costly, cumbersome, and not suitable for POC applications^7^. An alternative and intrinsically simpler approach is to exploit the phase sensitivity of gold-nanohole arrays^9^, which have shown LODs on the order of 10^−4^ RIU with a detection limit for proteins in tens of µg mL^−1^ range^10^. The goal of our work, which is targeted at POC applications, is to maintain simplicity while improving both the sensitivity and the dynamic range.

Regarding simplicity, most high-performance sensor modalities require expensive light sources, spectrometers, or other read-out instruments, and often need precise coupling arrangements. Dielectric metasurfaces and guided-mode resonances (GMRs) provide out-of-plane coupling, high sensitivity, and a wide field of view for multiplexed sensing. In particular, the chirped GMR approach^11^ translates spectra into spatial information to remove the need for a spectrometer. Following the same idea of translating spectra into spatial information, dielectric metasurfaces based on elliptical elements have recently been introduced^12,13^. These approaches both offer the necessary simplicity required for future miniaturization and the required sensitivity for many biosensing applications. Applying common-path interferometry^14^ with its intrinsic stability to such dielectric resonant structures can further reduce the limit of detection due to its sharp phase response and intensity noise-tolerant readout based on Fourier analysis.

The first proposal to explore the phase response of GMRs for sensing applications dates back to 2004^15^, where the rapid phase variation on resonance was studied by simulation, and an enhanced detection sensitivity was predicted for an interferometric read-out approach. The first experimental realization of this idea was not demonstrated until 10 years later, with a transmission-type GMR sensor in a heterodyne interferometer configuration^16^. The same group also investigated an approach based on phase-shift interferometry^17^. Both approaches require complex experimental equipment and phase-reconstruction methods. An alternative is to use an external Mach–Zehnder configuration^18^. The issue with the external Mach–Zehnder is its high sensitivity to environmental noise, however, rendering it unstable and unsuitable for POC applications.

To combine the simplicity of the GMR with the high sensitivity of interferometry while ensuring robustness and stability, we have developed a common-path interferometric approach by referencing two orthogonally polarized and independently optimized resonant modes of a dielectric nanostructure. By measuring the relative phase change between the two modes rather than the absolute change of an individual mode, our approach minimizes the impact of mechanical, thermal, and laser noise.

## Results

### Phase-sensitive detection principle

**Fig. 1:**
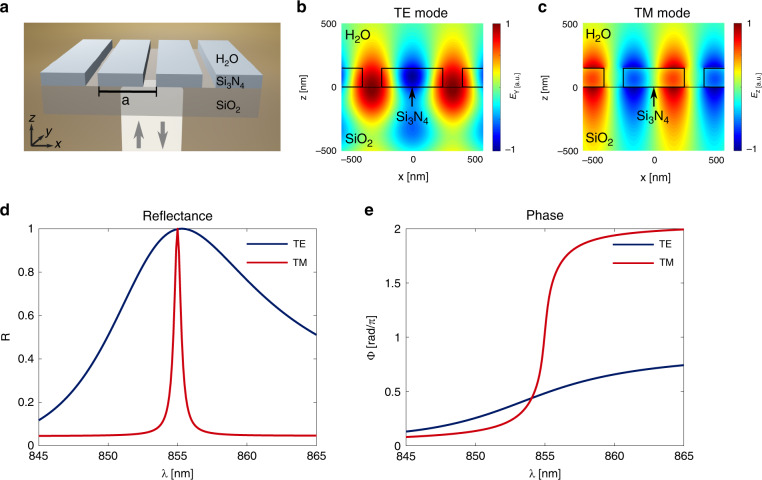
Schematic of the GMR structure and simulation results. **a** Schematic of a typical GMR structure consisting of a silicon nitride (*n* = 2) grating of 150-nm thickness on a glass (*n* = 1.45) substrate immersed in water (*n* = 1.33). The grating period in this example is *a* = 572 nm, and the filling factor is 75%. Collimated light is incident from the glass substrate layer. The TE/TM mode is excited when the E-field vector of the incident beam is oriented perpendicular/parallel to the grating vector. **b**, **c** Dominant E-field components of the TE (*y* component) and TM (*z* component) modes with an evanescent field decaying from the grating surface into the cover layer. See Supplementary S5 for more information on dominant field components. **d** Simulated reflectance with spectral mode overlap and **e** simulated phase response, using rigorous coupled wave analysis (RCWA)

GMRs can operate in transmission or in reflection. We have chosen to operate in reflection to separate the optical and electronic elements from the fluidic elements. This approach avoids the need to pass light through the bulk of the liquid, as operation in transmission would require, ensuring that the coherent light does not accumulate phase noise due to turbulence introduced by the microfluidic system. With these boundary conditions, a common-path interferometric approach can exploit the fact that these dielectric nanostructures support two modes of orthogonal polarization with different phase sensitivities^19^. We refer to the mode with the dominant electric field oriented along the grating grooves (*y* direction, Fig. 1) as the TE mode, and the mode with the dominant electric field perpendicular to the grating plane (*z* direction, Fig. 1) as the TM mode. The photon lifetime of the modes guided in a GMR grating is intrinsically limited as a result of scattering in the grating waveguide layer, leading to the nature of leaky modes^20^. Since resonance bandwidth and lifetime are inversely related^21^, a high-quality factor (Q factor) of the resonance peak corresponds to a higher phase sensitivity.

Rigorous coupled wave analysis (RCWA) simulations (Fig. 1d, e) illustrate the considerably higher Q factor and sharper phase response of the TM mode than of the TE mode. The lower Q factor of the TE mode can be explained by the field distribution shown in Fig. 1b. The TE mode can easily scatter into the far field because the optical fields at the Si_3_N_4_–H_2_O interfaces of the grating grooves are in phase. In contrast, the fields at the interfaces for the TM mode (Fig. 1c) are in antiphase, so scattering into the far field is suppressed by destructive interference.

**Fig. 2:**
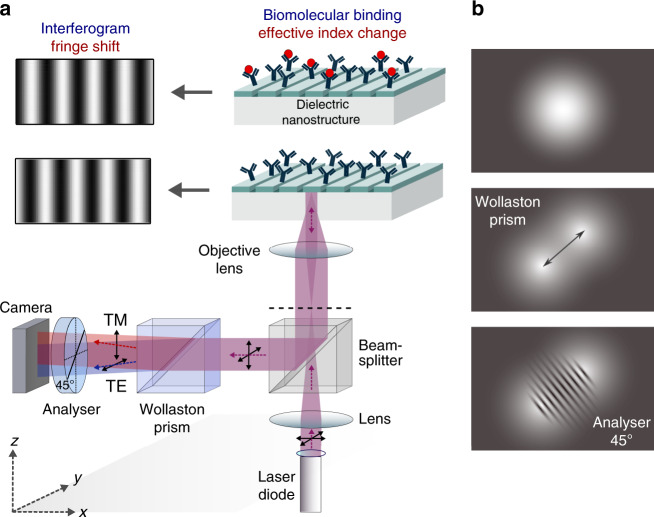
Schematic of phase-sensitive detection principle. **a** Optical setup where the output of an 855-nm laser diode is focused onto the back-focal plane of a 5× objective. This setup ensures that collimated light excites the guided-mode resonance. The polarization of the incident light is oriented at 45° with respect to the grating grooves, such that the TE and TM modes are equally excitable. The resonantly reflected light from the sensor carries information about the effective refractive index of the orthogonally polarized guided modes stored as phase information. The Wollaston prism introduces an angle of 1° between the two modes. **b** The two diverging beams overlap in the camera plane, and generate a high-contrast interferogram for an analyzer orientation of approximately 45°

The intrinsically high phase sensitivity of the TM mode (233 *π*/RIU for the structure simulated in Fig. 1) compared with 5 *π*/RIU for the TE mode enables them to be used as signal and reference modes, respectively. Creating an interferogram where the fringe position depends on the relative phase difference between the TE and TM modes then requires the introduction of an angle between the resonantly reflected, orthogonally polarized beams. We introduce a small angle of 1° by including a Wollaston prism into the output arm of the beam path (Fig. 2a). An angle of 1° is chosen to ensure adequate overlap of the sheared beams. An analyzer placed in front of a camera then projects both beams onto the same polarization state, which creates an interferogram in the beam-overlap area (Fig. 2b).

**Fig. 3:**
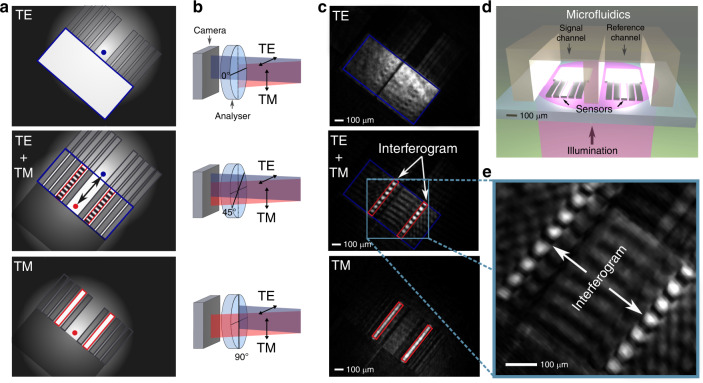
Schematics and camera images showing the spatial grating overlap principle. **a** Schematic of interferogram formation with spatial mode overlap of adjacent structures designed to resonate for TE/TM polarization shown for 0°, 45°, and 90° analyzer orientations. The blue/red frame indicates the structure resonating for TE/TM polarization. **b** Corresponding analyzer orientations and **c** corresponding camera images. The area with a blue frame is a uniform grating designed to resonate at *λ* = 855 nm in water for TE polarization. For details on nanostructure design, see Supplementary S6. Correspondingly, it shows high reflectance for the 0° analyzer orientation. The narrow stripes are designed, such that one resonates for TM polarization; since the TM resonance is much narrower, we use several stripes consisting of gratings with different periods (Δ*a* = 1 nm) to ensure that at least one of them is on resonance within the expected refractive index range of the analyte. This principle ensures a high dynamic range. Correspondingly, only one of the grating stripes lights up at the 90° analyzer orientation (red frame). When the analyzer axis is set to 45°, the TE and TM components can interfere and create an interferogram in the beam-overlap region (see also Fig. 2). **d** Schematic of sensors integrated in a microfluidic system. We design a signal and a reference channel to enable drift subtraction. **e** Zoom of **c** (middle)

The most obvious way of realizing this common-path interferometric approach would be to design a single grating, such that both modes overlap spectrally. While this is possible (Fig. 1d), it is only achievable for a restricted set of grating parameters (Supplementary Fig. S3), and would therefore overly constrain the ability to optimize the sensing performance, particularly the phase sensitivity. Especially for high grating fill factors, corresponding to a higher phase sensitivity, the TE and TM modes do not overlap spectrally at all. This fact can be explained by the TE mode being predominantly confined to the grating grooves (Fig. 1b), and therefore being more sensitive to changes in the grating fill factor. Alternatively, two separate gratings with independently optimized grating parameters can be designed to ensure resonance at the same wavelength for both polarizations. The reflected signals from the two gratings are then spatially superimposed in the camera plane, as illustrated schematically on the bottom image of Fig. 2b and demonstrated experimentally in Fig. 3c.

The principle of interferogram formation based on spatial mode superposition is demonstrated in Fig. 3 by showing the same image with three different analyzer orientations with the Wollaston prism axis being fixed, such that the TE- and TM-polarized beams diverge at an angle of 1°. For an analyzer orientation of 0°, the light reflected from the grating designed to resonate for TE polarization passes through the analyzer, while for an orientation of 90°, only the TM resonance is visible in the adjacent grating. A grating length of 500 µm is chosen to ensure the spatial TE and TM mode overlap in the image plane. We note that the fringe contrast in the beam-overlay area is dependent on the intensity ratio between both resonances, and can be optimized via the analyzer orientation. Using separate grating parameters for each polarization ensures that the resonance wavelengths overlap for both modes, and that both gratings can be optimized independently for their purpose as the origin of signal and reference beams, respectively (further explanation of independent TE and TM mode optimization is provided in Supplementary S3).

The dynamic range of the measurement is generally limited by the sharper resonance, here, the TM mode. Since the maximum phase sensitivity occurs around the center of the resonance, both the sensitivity and the contrast of the interferogram decrease as we go through the resonance. Clearly, the sharper the TM resonance is, the more sensitive the measurement and the smaller the dynamic range. To overcome this limitation, we design several grating stripes of slightly different periods for the high-Q TM mode. Having multiple stripes probing adjacent phase curves improves tolerances against fabrication, temperature, and incident angle variations, as well as broadens the range of available biosensing applications. Providing multiple stripes is only required for the TM mode since the TE mode resonance is substantially broader and therefore intrinsically more tolerant. To also account for any system drift, which may be caused by temperature variations in either the environment or the analyte, we further implement a differential scheme using a second interferometric GMR as a reference channel (Supplementary Fig. S1). The reference channel is isolated from the signal channel via a microfluidic manifold, and the refractive index in the reference channel is kept constant, such that it can be subtracted from the signal channel to yield the effective phase response.

### Experimental phase sensitivity and refractive index limit of detection

We first perform bulk sensitivity and noise measurements to characterize the refractive index LOD of the sensor before moving on to protein sensing.

**Fig. 4:**
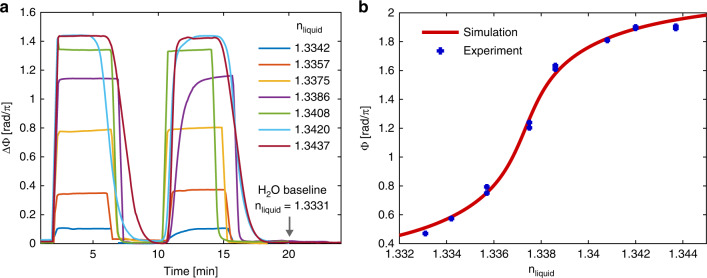
Bulk sensitivity measurements and experimental phase curve. **a** Starting from a H_2_O baseline with a refractive index of 1.3331, glucose solutions of a higher refractive index in a range between 1.3342 and 1.3437 were flowed over the surface, and the corresponding phase response was measured. The measurement was repeated twice for each concentration to verify system stability. **b** Phase curve obtained from the measurement on the left in comparison with the simulated phase response. Note the good agreement between the simulation and experiment, as well as the good repeatability of multiple measurements. For further discussion of accuracy limitations, see Supplementary S1

The bulk sensitivity is determined by challenging the sensor with glucose solutions of stepwise- increasing refractive index (calibrated with a refractometer), and measuring the corresponding phase response (Fig. 4). We use independently optimized TE and TM modes of adjacent structures (see Fig. 3) in order to increase the filling factor for higher phase sensitivity of the TM mode (Supplementary S3). In the most sensitive range of the corresponding phase curve, synonymous with the highest reflectance, we measure a sensitivity of *S* = 289 *π* RIU^−1^. Because we now independently optimize the TM mode (filling factor of 80%), the obtained sensitivity is higher than 233 π RIU^−1^ (filling factor of 75%, Fig. 1). In combination with the measured 3*σ* noise level of 5.4 × 10^−4^ *π* (Supplementary S1), we obtain a bulk limit of detection of LOD = 3*σ*/*S* = 1.8 × 10^−6^ RIU. Since our sensor is targeted at POC applications, we characterized the noise over a 30-min time interval, approximately twice the time to reach saturation at low concentrations of biomarkers.

### Extension of the dynamic range

**Fig. 5:**
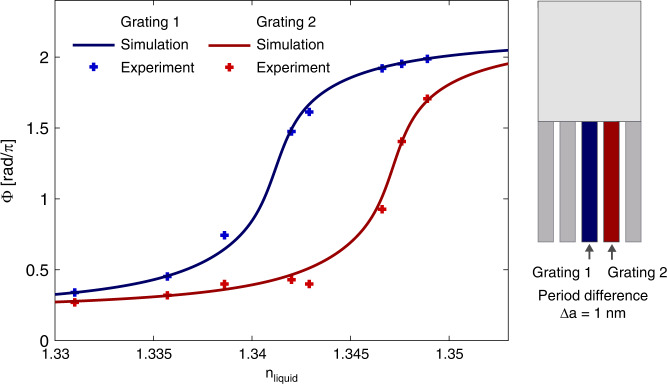
Experimental demonstration of the extended dynamic range principle. Implementation of several grating stripes with a difference in period of Δ*a* = 1 nm (schematic on the right). The two phase curves were obtained in the same way as in Fig. 4 with glucose solutions in the index range between 1.3331 and 1.3489. For design details, see Supplementary S6

The extension of the dynamic range is illustrated in Fig. 5, which shows the phase curves of adjacent gratings with a grating period difference of 1 nm. This difference in grating period was chosen to ensure a seamless transition between adjacent resonances. For biosensing applications, a wide dynamic range provides several important advantages. First, it ensures tolerance to environmental conditions, such as temperature and incidence angle, as well as fabrication tolerance. In terms of POC biosensing applications, the dynamic range ensures a versatile application range with clinical relevance by enabling both the detection of low concentrations of small protein biomarkers at the onset of disease, which we are demonstrating in this work, and the detection of higher concentrations and larger biomarkers or protein aggregates, including monitoring the personalized response to treatments. Here, we show a dynamic range of Δ*n* = 0.017 RIU, which is 4 orders of magnitude above the LOD, and can be extended further by considering more grating stripes, if required.

### Protein sensing

To characterize the protein detection performance of our sensor, we have chosen to sense PCT that is synthesized in the human body in response to the invasion of pathogenic bacteria, and has been recognized as an early and specific indicator of bacterial infection^2^. As PCT can be used to differentiate between bacterial and viral infections, it thus has the potential to inform the appropriate use of antibiotics. Despite being a promising biomarker, PCT is not used widely in label-free POC devices because it occurs at low concentrations and is relatively small (13 kDa). A potential POC device for PCT needs to provide an LOD of tens of pg mL^−1^, which is challenging, especially for label-free detection. Using our highly sensitive photonic technology, we are able to detect PCT down to a concentration of 1 pg mL^−1^ in an entirely label-free fashion. We note that the observed 0.019 π phase shift for this concentration is well above the noise level (SNR = 35), which means that even lower LODs are possible. The closest comparison that we could find for PCT is a nanoplasmonic system that uses labels and achieves an LOD of 21 pg mL^−1^, albeit in a portable system and with clinical samples^22^.

**Fig. 6:**
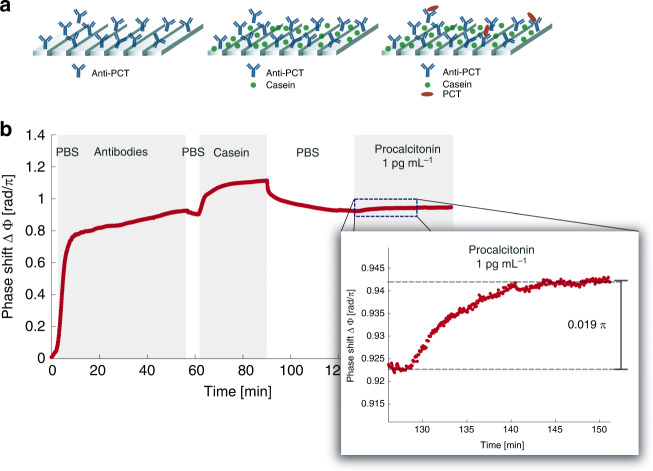
Protein sensing. **a** Schematic of functionalization steps and the corresponding phase shift observed experimentally in (**b**); anti-PCT antibodies (50 µg mL^−1^) are binding to the PEG spacer layer (see “Methods”). After the surface is saturated, casein is introduced to block any remaining nonspecific binding sites. The subsequent PBS washing results in most of the casein being washed away, suggesting that the PEG layer effectively blocks the surface. We observe a 0.019π phase shift in equilibrium for a procalcitonin concentration of 1 pg mL^−1^. The Langmuir isotherm in Fig. S4 confirms that we observe actual binding to the surface-immobilized antibodies

As shown in Fig. 6, introducing a casein blocker results in only a small and mostly reversible phase shift, which suggests that the well-known PEG chemistry^23,24^ (Methods) effectively inhibits nonspecific protein adsorption. The lack of nonspecific binding is an essential requirement for the analysis of clinical samples, which we will implement in future work. We also note that the binding curve for 1 pg mL^−1^ follows the expected Langmuir isotherm typical of antibody–ligand association (Supplementary Fig. S4), which confirms that the observed phase shift is not due to simple physisorption of proteins.

## Discussion

Our combination of common-path interferometry and GMRs has enabled us to demonstrate a technology that meets the main requirements for a label-free photonic biosensor for applications at the POC: it achieves an ultralow limit of detection for biomolecular binding events, a wide dynamic range for high tolerance, and versatile clinical use, including personalized medicine, as well as multiplexing capability and simplicity of operation and setup.

The ultralow limit of detection is facilitated by the sharp phase response of GMRs observed at the point of maximum field enhancement along with the readout enabled by interferometry. We introduced the novel concept of spatially superimposing two beams carrying the phase information of two independently excited resonant modes with different phase sensitivities, and interferometrically measuring the relative phase difference between them. This idea can be applied to other resonant nanostructures and metamaterials. For example, by using higher refractive index materials, a higher surface sensitivity and thus an even lower limit of detection for biomolecules could be achieved in the future. We note that the application of our idea does not require form birefringence of the resonant structure, meaning that nanostructures with two-dimensional symmetry can also be used, which further adds to the versatility of our approach.

Additional capabilities can be added because of the large field of view of our system. This capability will allow us to readily implement additional antibody channels for multiplexing, thus offering the ability to detect multiple biomarkers in parallel.

Importantly, the demonstrated ultralow limit of detection for biomolecules does not rely on an actively stabilized laser source or a stabilized environment. Because of the common-path interferometric approach and the differential drift compensation scheme, the sensor can be operated in an unstable environment with a low-cost laser diode source and a camera for the readout, rendering the technology intrinsically simple and suitable for mass-market applications.

## Methods

### Sensor chip fabrication

We use commercial wafers consisting of a 150-nm thick film of Si_3_N_4_ on a 500-µm glass substrate (Silson, UK) and dice them into (15 × 15) mm^2^ pieces before cleaning them by sonication in acetone (ACE) for 10 min, rinsing in isopropanol (IPA), and placing in a plasma asher for 5 min. The resist that we use for electron-beam lithography (EBL) is AR-P 6200.13 from Allresist GmbH spun at 5000 rpm for 60 s and baked on a hotplate at 180 °C for 5 min. For charge dissipation during EBL exposure, we use the conductive polymer AR-PC 5090 (Allresist GmbH) spun at 2000 rpm and baked on a hotplate at 90 °C for 2 min. The pattern is written with a Voyager EBL system from Raith GmbH, 50 kV, with a beam current of 130 pA and a dose of 150 µC cm^−2^. After removing the charge dissipation layer in deionized water at room temperature for 2 min, we develop the pattern in xylene for 2 min at room temperature, and stop the development with a rinse in IPA. Next, the pattern is transferred into the Si_3_N_4_ layer by plasma-based reactive ion etching (RIE) using a gas mixture of O_2_ and CHF_3_ at a ratio of 2:58. After etching for 7 min, we remove the remaining resist with gentle sonication in a 1165 Microposit Remover (Shipley) for 10 min.

### Microfluidic system fabrication

To fabricate a mold for the polydimethylsiloxane (PDMS) channels, we first clean a silicon wafer piece with sonication in ACE and a rinse in IPA and deionized H_2_O. We spin the permanent epoxy-negative photoresist SU-8 2050 (Microchem Inc.) at a speed of first 250 rpm for 10 s, then 750 rpm for 10 s, and finally 1000 rpm for 60 s to reach a thickness of ~170 µm, and we soft-bake at 65 °C for 5 min and then 95 °C for 30 min. Next, we expose the patterns with direct laser writing (KLOÉ DILASE, *λ* = 375 nm) with a 10× objective, 1 mm s^−1^ stage speed, and 65% modulation. Following exposure, we post exposure bake at 65 °C for 5 min and 95 °C for 12 min. Then, we develop the pattern in EC solvent at room temperature for 15 min with constant agitation. We stop the process with a rinse in IPA, and leave the sample in an oven at 180 °C overnight. The PDMS is created by mixing elastomer and curing agent at a ratio of 7:1 and leaving it in a desiccator for 20 min to remove air. This mixture is then poured over the channel mold and baked at 60 °C overnight. We remove the PDMS from the mold, and use a micropuncher to create holes for the microfluidic tubing.

### Surface chemistry

We first clean the sensor surface in Piranha solution, which consists of a mixture of sulfuric acid and hydrogen peroxide at a ratio of 21:9. This step also ensures hydroxylation of the sensor surface, which is necessary for the salinization step following cleaning. To salinize the Si_3_N_4_ surface, we leave it in a 5% solution of (3-mercaptopropyl)trimethoxysilane (MPTS) in dry ethanol for 6 h. Following a rinse in ethanol and in dimethyl sulfoxide (DMSO), we functionalize the surface overnight with a PEGylated SMCC cross-linker (SM(PEG)6 from Thermo Fisher) at a concentration of 1 mM in DMSO while avoiding exposure to humidity during this process. Following a rinse in DMSO and drying the sensor with nitrogen, we clamp it to a microfluidic channel made of PDMS, and the remainder of the surface functionalization protocol takes place in this microfluidic system at room temperature. We use a microfluidic pump to pull the solutions over the surface through the microfluidic channels at 20 µL min^−1^. To avoid hydrolysis, we introduce a short PBS baseline for 10 min before flowing the anti-PCT antibody (Abcam) at a concentration of 50 µg mL^−1^ in PBS over the sensor surface for 1 h. Then, a 10-min PBS washing step is introduced before flowing a 1% casein-blocking buffer (diluted 10X casein- blocking buffer from Sigma-Aldrich, UK) for half an hour to ensure that any remaining nonspecific binding sites are blocked before the final target biomarker binding. The PBS washing step after casein is continued, until a flat phase response is reached, which takes 30–45 min. Then, PCT (recombinant human PCT protein, Abcam) in PBS is introduced to the functionalized sensor surface.

### Optical setup and data acquisition details

We use an inverted microscope for sensing experiments in reflection, with only a few modifications necessary for phase-sensitive operation. These modifications include using a coherent source, and implementing a Wollaston prism and analyzer. The collimated output of a VCSEL-based laser diode module (Thorlabs Inc., CPS850V) is directed toward a lens and focused in the back-focal plane of a 5X objective. The objective collimates the light, and after being resonantly reflected from the GMR sensor, the beam is directed toward the Wollaston prism (Thorlabs Inc., WPQ10) by a beam splitter. The Wollaston prism is aligned and fixed, such that the orthogonally polarized TE and TM modes are split into two diverging beams with approximately equal intensity. The analyzer then enables the common components of these beams to interfere, and the interferogram is recorded with a CMOS camera (Thorlabs Inc., Compact USB 2.0 CMOS Camera). The contrast of the interferogram fringes can be adjusted in the beginning of a measurement by rotating the analyzer. For sensing experiments, we take images at a rate of 0.2 fps. Fast Fourier transform-based post processing is performed with a custom MATLAB script.

## Supplementary information

Supplementary Information

## Data Availability

The datasets generated during and/or analyzed during the current study are available from the corresponding author on reasonable request.
