# Supplementary material for: Common-path interferometric label-free protein sensing with resonant dielectric nanostructures
Source: Light Sci Appl. 2020 Jun 2;9:96. doi: 10.1038/s41377-020-0336-6 (PMC7264974; doi:10.1038/s41377-020-0336-6)
Supplement: Supplementary file 1 — Supplementary Information [file 41377_2020_336_MOESM1_ESM.docx]

#

# **Common-path interferometric label-free protein sensing with resonant dielectric nanostructures**

Supplementary Information

# Isabel Barth^1^*, Donato Conteduca^1^, Christopher Reardon^1^, Steven Johnson^2^ and Thomas F. Krauss^1^

^1^Department of Physics, University of York, YO10 5DD York, UK

^2^Department of Electronic Engineering, University of York, YO10 5DD York, UK

*Email: [isabel.barth@york.ac.uk](mailto:isabel.barth@york.ac.uk)

*Phone: [+44(0)1904 322699](tel:+44%201904%20322699)

[donato.conteduca@york.ac.uk](mailto:donato.conteduca@york.ac.uk)

[christopher.reardon@york.ac.uk](mailto:christopher.reardon@york.ac.uk)

[steven.johnson@york.ac.uk](mailto:steven.johnson@york.ac.uk)

[thomas.krauss@york.ac.uk](mailto:thomas.krauss@york.ac.uk)

# **S1. Bulk limit of detection with microfluidic referencing system**

In order to determine the bulk limit of detection (LOD), we characterize the system noise in addition to the sensitivity (manuscript Figure 4). In order to account for system drift, potentially caused by laser diode wavelength and environmental temperature drift, we implement differential microfluidic referencing for compensation of these noise introducing factors. This is realized in the same field-of-view by adding a mirrored copy of the nanostructure to the reference channel (Figure S1a).


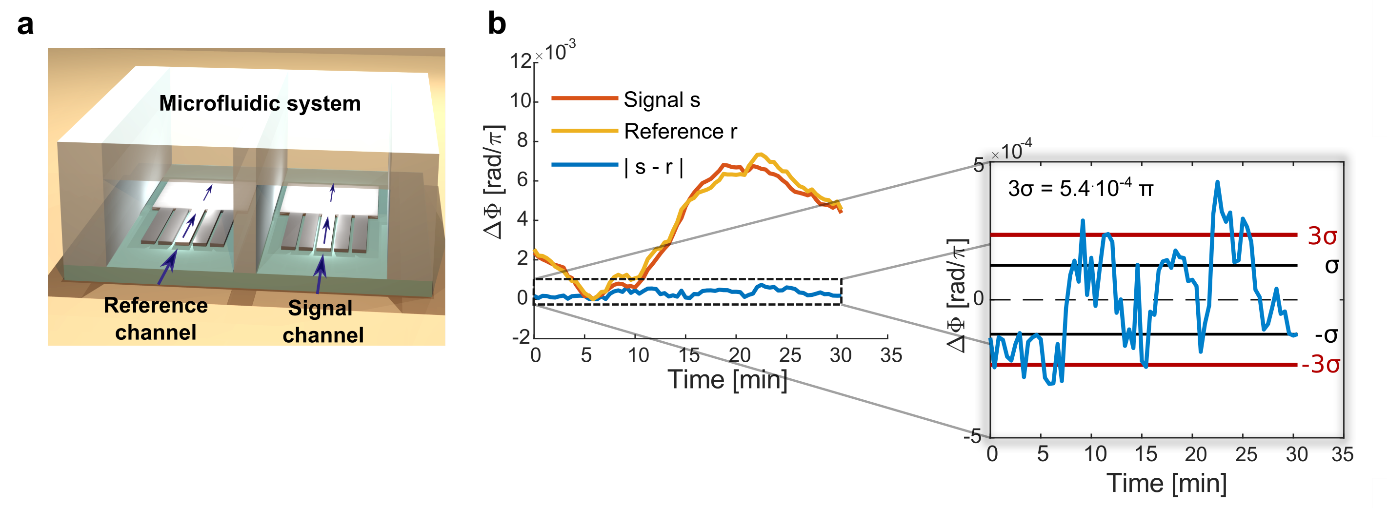
***Figure S1*** *-* ***Noise characterization for LOD determination. a,*** *Schematic of microfluidic referencing system.* ***b,*** *Noise characterization for LOD determination with referencing system*

# We characterize the noise by measuring the standard deviation of the phase response while flowing deionized water for 30 minutes. The measured signal *s* and reference *r* phase responses are plotted in Figure S1b as well as the effective phase response, which is obtained by calculating |s-r|. The resulting standard deviation (here expressed as 3σ) of this effective phase response is 3σ = 5.4∙10^-4^ π. The remaining noise, after referencing, is not statistical, which means low pass filtering does not lower the 3σ value.

In order to further reduce the noise level, an equal phase sensitivity in signal and reference microfluidic channel would be required. This can be reached by removing slight angular excitation differences in the field-of-view, which can occur due to the incident beam being not perfectly collimated.

# **S2. SEM, AFM and phase contrast microscopy images of fabricated nanostructure**

We analyze the fabricated nanostructures with scanning electron microscopy, atomic force microscopy and phase contrast microscopy, shown in Figure S2a. These images are included here to give a clear visualization of the dimensions and quality of the structures, which are used for the experiments described in the manuscript.

***
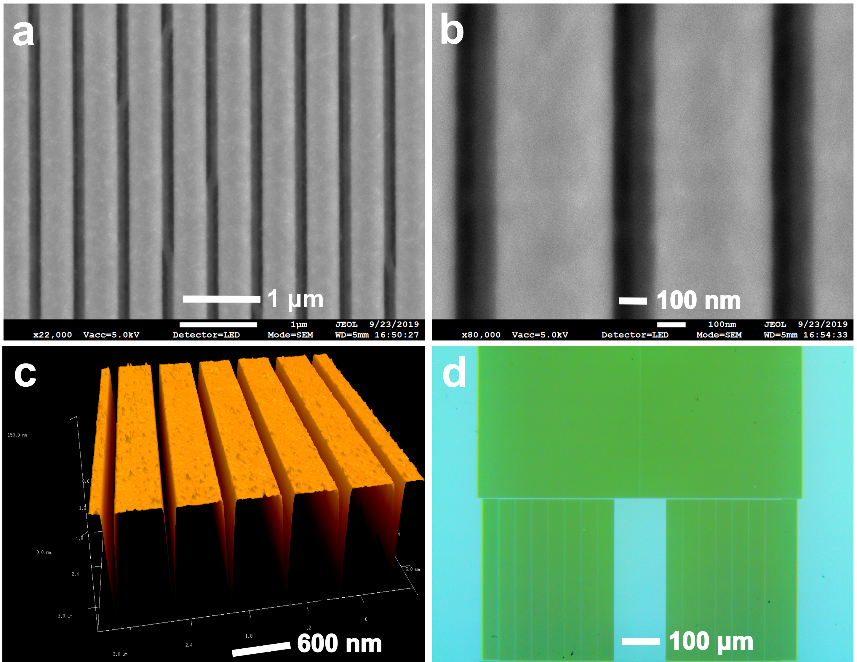
***

***Figure S2. a****, SEM micrograph of grating structure after resist removal and* ***b****, magnification of a.* ***c****, AFM of grating structure,* ***d****, Phase contrast microscope image of the entire structure, including two identical copies (left, right) for the signal and reference microfluidic channels. The large block at the top is the grating designed to resonate for the TE mode while the bottom section of the image shows the grating stripes (here 8 stripes with grating period difference 0.5 nm) designed to resonate for the TM mode.*

**S3. Motivation for independent TE and TM mode optimization**

To further clarify the motivation behind the independent optimization of the TE and TM gratings, we show a rigorous coupled-wave analysis (RCWA) simulation in Figure S3. Since the quality factor of the guided-mode resonance increases with the effective index of the mode, a high grating filling-factor is desirable for maximized sensitivity. As mentioned in the manuscript, the TE mode is more sensitive to fill-factor changes than the TM mode because it is predominantly confined to the grating grooves. For example, the TE and TM modes spectrally overlap perfectly in the case of a fill-factor of 75% while the overlap is close to zero for a fill-factor of 80%, where the TM mode has its highest phase sensitivity.


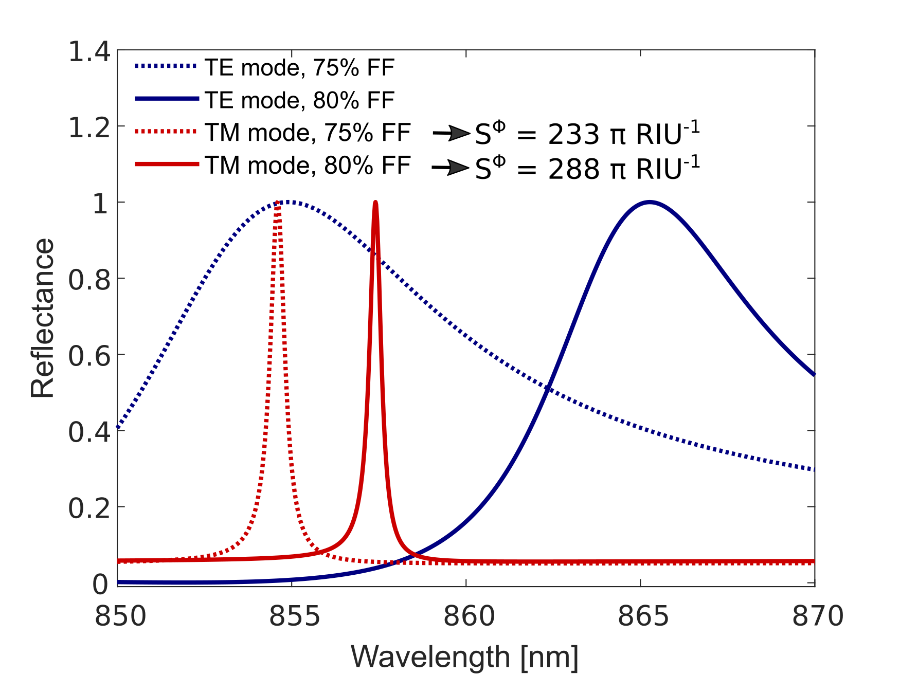


# ***Figure S3 - RCWA simulation on restricted spectral overlap of TE and TM mode****. The dashed TE and TM spectra overlap perfectly when the fill factor (FF) is chosen to be 75% Si_3_N_4_ whereas the modes do not overlap at the same grating period of 572 nm with FF = 80%. Choosing a higher FF for the TM mode is advantageous because a higher effective index results in higher phase sensitivity. For this reason, we choose to design independent gratings for TE and TM mode to ensure a respective resonance at the laser wavelength. In this way, we do not rely on spectral mode overlap and can optimize the TE and TM gratings for their respective purpose as reference and signal beam.*

Following this example, in order to improve sensitivity, we would need to measure the phase difference between two nanostructures, which are optimized for their respective purpose as signal (TM mode, high sensitivity) and reference (TE mode, low sensitivity) without the need for spectral mode overlap. We achieve this by designing independently optimized, adjacent gratings with a high filling-factor for the TM grating and then creating an adequate shear between the resonantly reflected TE and TM polarized light to achieve spatial TE/TM overlap. In this case, the TE and TM modes of the same structure would be spectrally separated and could not interfere. Designing the two gratings separately adds versatility and the ability to optimize both gratings independently.

# **S4. Procalcitonin binding characterized by Langmuir Isotherm**

Figure S4 shows that procalcitonin (PCT) binding to the corresponding antibody on the sensor surface follows the expected Langmuir trend of antigen-antibody association. By following the Langmuir binding curve, we ensure that we observe actual binding rather than simple physisorption.


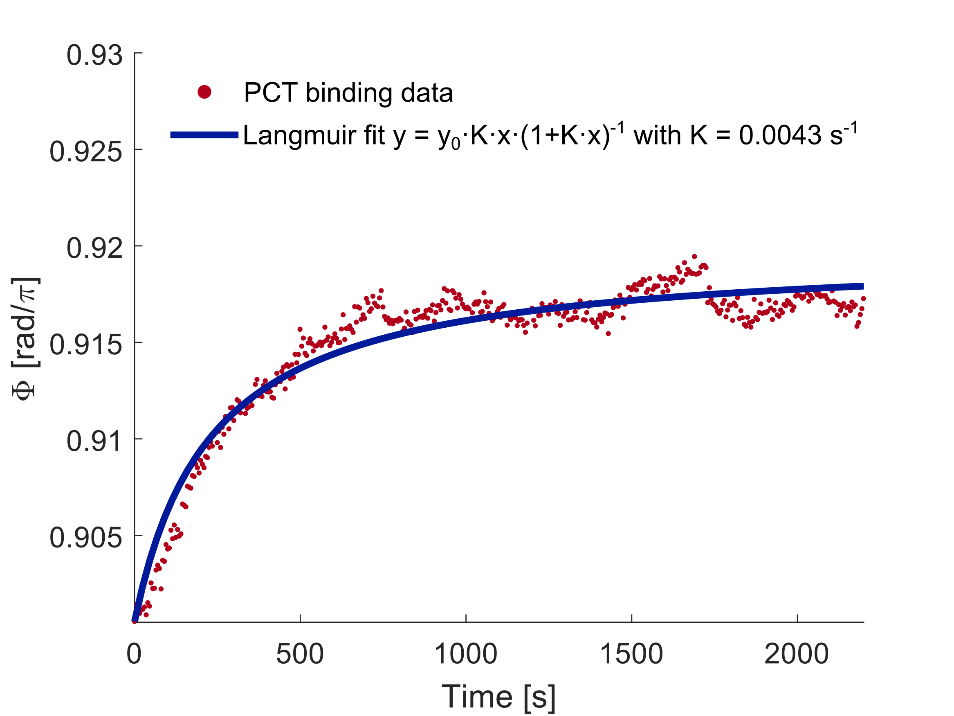


# ***Figure S4 - Binding of procalcitonin characterized by Langmuir isotherm.*** *Langmuir isotherm fitting of PCT (1 pg mL^-1^) association demonstrates that the PCT binding follows the expected trend of antibody-ligand association. K represents the on-rate of the association.*

# *.*

# **S5. Dominant Electric Field components of TM mode**

This section aims to explain Figure 1b and c in the main manuscript in more detail. A guided-mode resonance is here observed when the first diffraction order of the grating structure is laterally guided in the grating layer. This means that normally incident light is diffracted and guided along the x-direction, which adds a z-component to the confined E-field in the case of the TM mode, whereas the polarization of the TE mode (in y-direction) remains the same. Figure S5 shows that the TM mode also still has an Ex component which is less dominant than the Ez component. This becomes apparent when comparing the Ex and Ez component to the total field |E|.


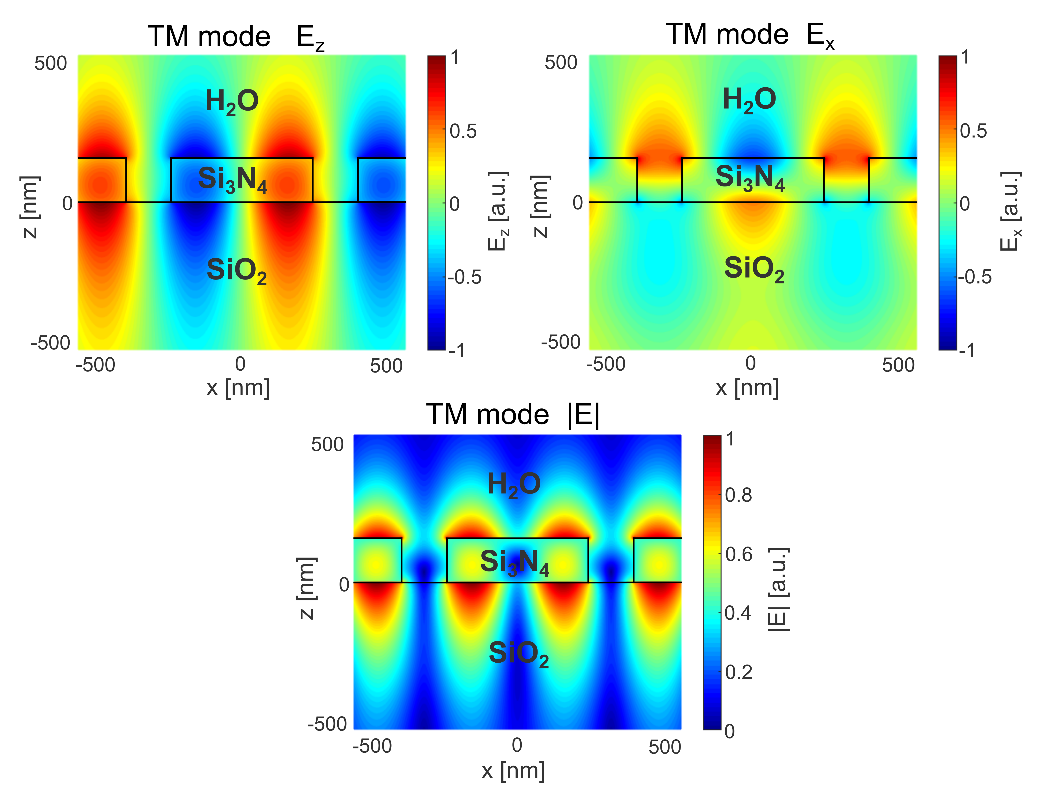


# ***Figure S5 - S^4^ simulations of TM field components at resonance.*** *The TM has an E_z_ as well as an E_x_ component (upper figures). When looking at the total field (*|*E*| *in the bottom figure*), *it becomes apparent that the E_z_ component is the dominant component.*

# **S6. Dielectric nanostructure design parameters**

The design of the dielectric structures in the field of view are shown in Figure S6. For all simulations we assume a SiO_2_ substrate refractive index of 1.45, a Si_3_N_4_ refractive index of 2.00 and a H_2_O cover refractive index of 1.33. The angle of excitation is zero. The two identical (350 x 500) µm^2^ structures (blue) on the top are designed to resonate for those components of the incident light which are polarized in direction of the grating grooves at the laser diode wavelength of 855 nm. A grating period of 580 nm in combination with a filling factor of 68% provides this resonance condition for the TE mode. The distance between the two identical structures is 200 µm, which is the distance of the microfluidic signal and reference channel.


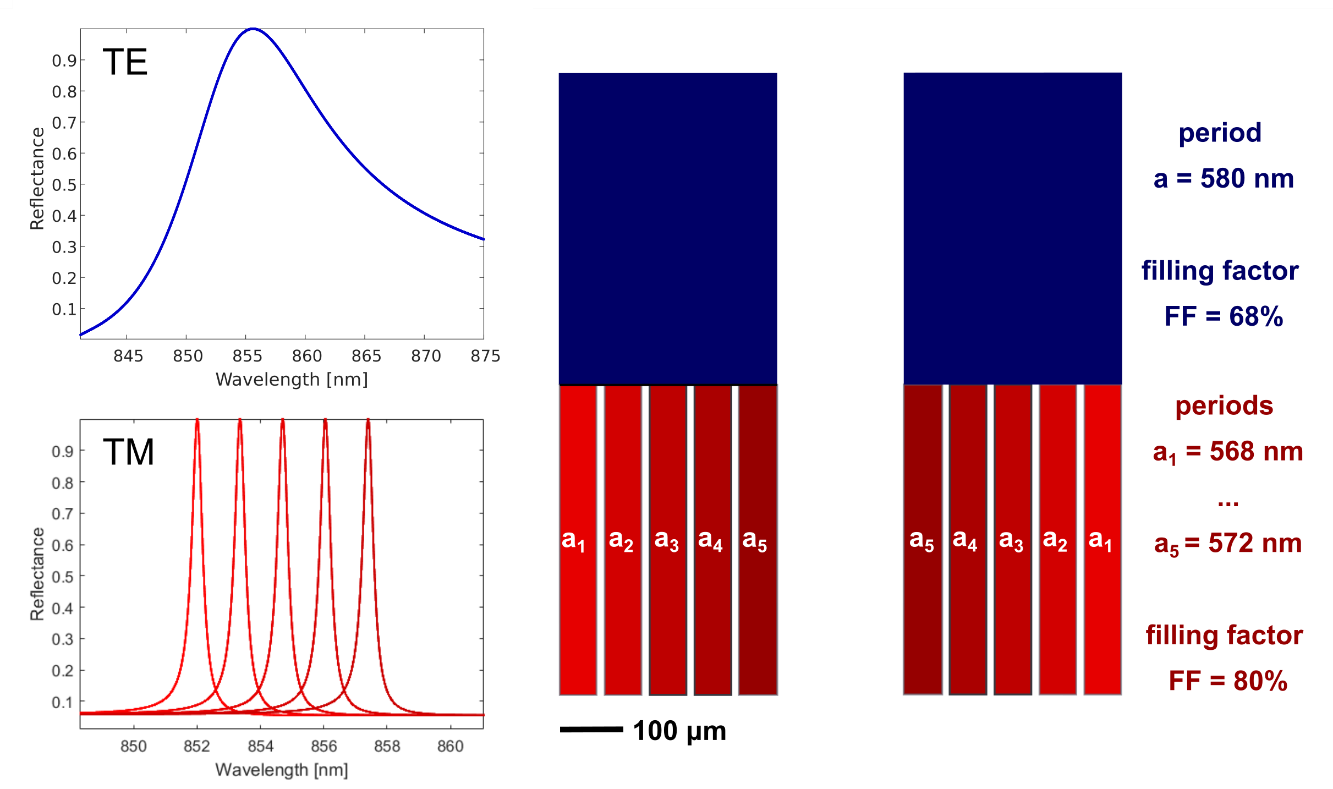


# ***Figure S6 - Design parameters and corresponding simulated resonance peaks.*** *Areas designed to excite a TE mode/TM mode are marked blue/red.*

In the bottom row, we show the implementation of five grating stripes in each channel. The grating period of these structures vary from 568 nm to 572 nm with a period step size of 1 nm in the left channel and a mirrored version (for symmetric excitation) of these stripes in the right channel. In combination with a filling factor of 80%, these parameters ensure a resonance wavelength around 855 nm for incidence light which is polarized orthogonal to the grating grooves (TM mode).

There is a trade-off between the width of each stripe (here 62 um), which allows for averaging and thus noise reduction, and the dynamic range, considering that we have a limited field of view available. The best choice of size and number of stripes depends on the specific application of the sensor. For our goal of measuring low concentrations of small proteins, a minimum of 5 grating stripes gives enough fabrication and incidence angle tolerance to ensure that a resonance from one of the stripes is visible at the operating wavelength. If, for example, a different application would require a wider dynamic range or higher tolerance, then a smaller grating width and a larger number of gratings could be implemented.

# **S7. Spectra of adjacent TM mode structures**

In order to investigate the spectral characteristics of the adjacent TM modes and the corresponding fabrication tolerance we carried out hyperspectral imaging using a combination of Halogen source and monochromator in order to obtain the high-resolution spectra in Figure S7. As expected, when comparing the response of two different structures (on the right), the central wavelength of the resonance peaks can shift slightly, whereas the FWHM (0.8 nm) and the distance between two adjacent peaks (1.4 nm) stays the same.


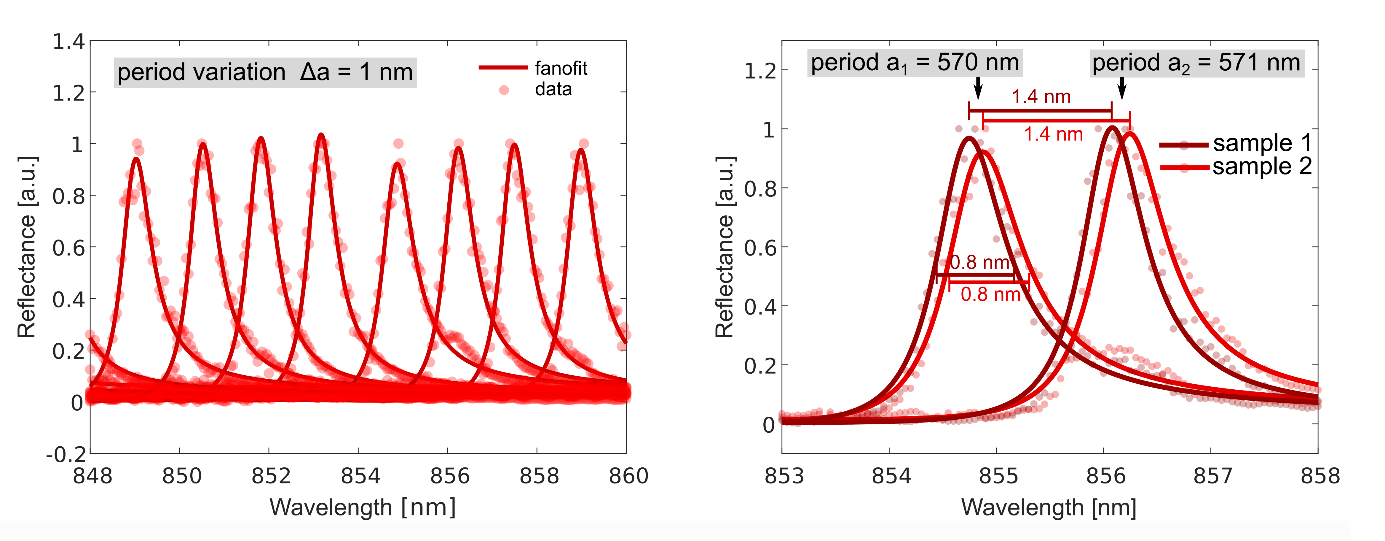


***Figure S7 - Spectra of adjacent TM structures with 1 nm period variation.*** *On the left, the spectra of structures in grating period range of 8 nm are shown. On the right, a zoom into two adjacent spectra is shown for two different fabricated samples. For both samples, the period of the left peak corresponds to a grating period of 570 nm and the right peak to a period of 571 nm. Both peaks of both samples show the same (within the error of the measurement) spectral characteristics.*

# **S8. Sensitivity compensation for linear extended dynamic range**

A grating period variation for an extended dynamic range provides a resonance wavelength compensation, meaning that at the single laser wavelength, an increase in refractive index leads to a resonance of structures with lower grating periods. Since the refractive index – resonance wavelength dependence as well as the period – resonance wavelength dependence is approximately linear (Figure S9), this compensation is straightforward. Nevertheless, as apparent in Figure S9, a period variation is not able to compensate for the change in spectral characteristics of the resonance when increasing the refractive index. This can be achieved by a combined period and filling factor variation.


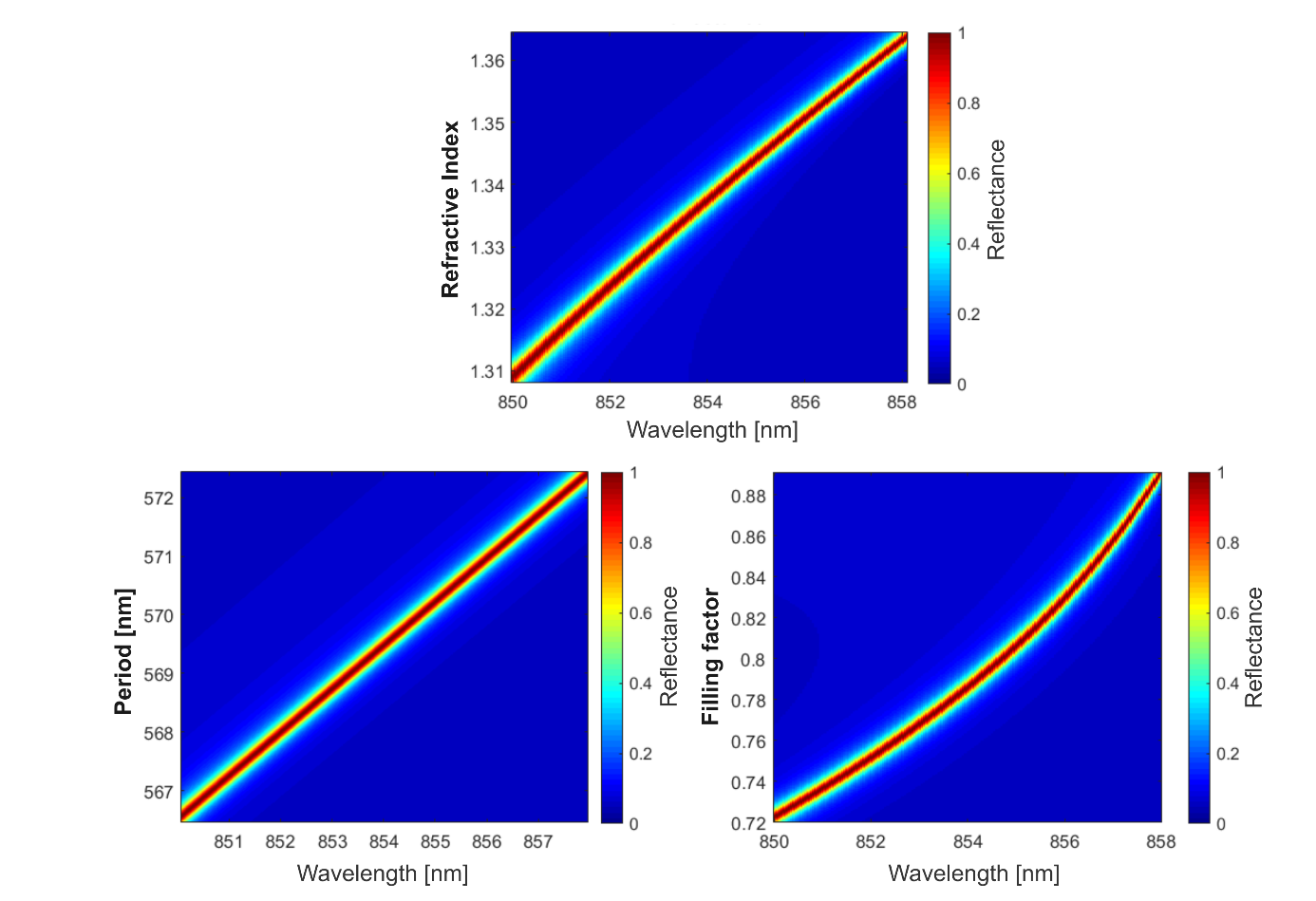


***Figure S8 - Resonance maps showing resonance wavelength dependence and spectral characteristics of TM mode.*** *The top map shows the resonance wavelength dependence on the bulk refractive index of the medium above the grating surface. The increase in Q-factor with index is also apparent. The left bottom map shows a linear response of the resonance wavelength with grating period variation. The Q-factor of the resonance peak is not influenced by the period. The right bottom map shows that the response of the resonance wavelength to changes in filling factor is not linear and that the Q-factor increases with higher filling factors.*

As an example, a change in refractive index of Δn = 0.03 would lead to an approximate FWHM decrease of 0.2 nm, corresponding to a 40% increase in phase sensitivity. Since a period variation does not influence the phase sensitivity of the resonant mode, this would thus lead to a non-linear dynamic range. Instead, the same phase sensitivity can be achieved for the same index change, if the filling factor is reduced from 80% to 74% while the period is reduced by only 1 nm to ensure the same resonance wavelength. In this manner, a dynamic range with constant phase sensitivity can be reached if the application requires a quantification of biomaterial on the surface over a refractive index range which exceeds the range of a single mode.
